# Supplementary material for: Lactobacillus acidophilus C4 ameliorates constipation in mice
Source: Microbiol Spectr. 2026 May 12;14(6):e02950-25. doi: 10.1128/spectrum.02950-25 (PMC13228036; doi:10.1128/spectrum.02950-25)
Supplement: Figures S1 and S2 — Figure S1: Fecal Lactobacillus colony counts in various treatment groups in mice. Figure S2: L. acidophilus C4 alleviates constipation-associated disorders of lipid metabolism levels. [file spectrum.02950-25-s0001.docx]

**
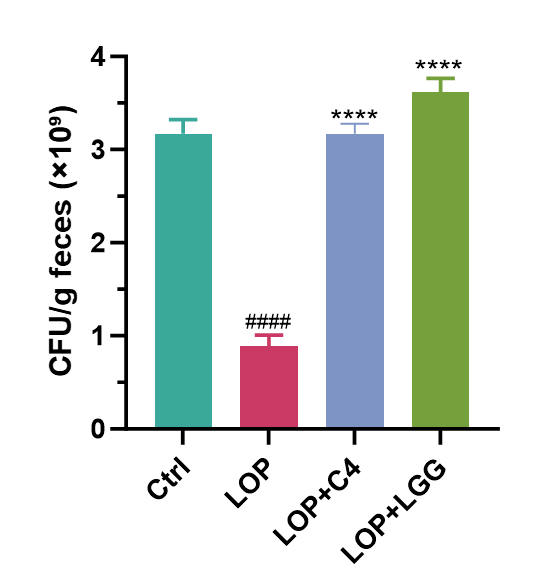
**

**Supplementary Figure S1 Fecal Lactobacillus colony counts in various treatment groups in mice.** At the experimental endpoint, freshly excreted feces from mice were serially diluted and spread onto bacterial culture plates, which were then anaerobically incubated for 24 hours prior to colony counting. Subsequent experiments included: (A) Colony counting and photography of bacterial culture plates; (B) Statistical analysis. Ctrl: Normal control group. LOP: Loperamide-induced model mice. LOP+C4: Loperamide-induced model mice prevented with *L. acidophilus* C4. LOP+LGG: Loperamide-induced model mice prevented with *L. rhamnosus* LGG. Data are expressed as mean ± standard deviation (SD) (n=9). Compared with the loperamide-induced model mouse group (****) *p* < 0.0001, compared with the normal control group, (####) *p* < 0.0001, is significant.


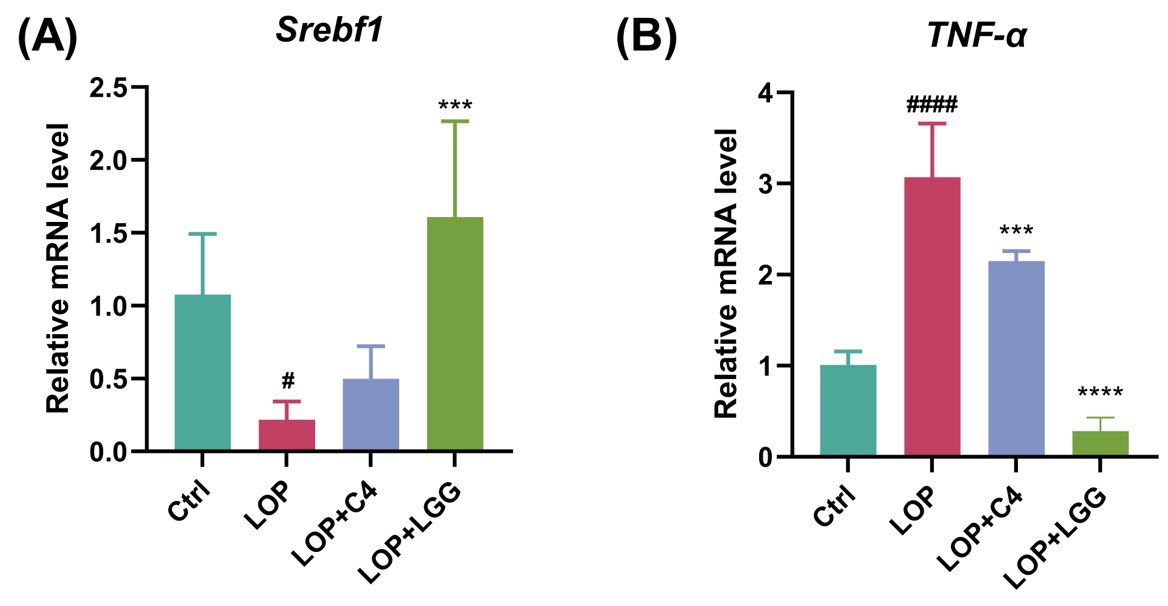


**Supplementary Figure S2 *L. acidophilus C4* alleviates constipation-associated disorders of lipid metabolism levels.** Mice were treated as Fig 1B, on day 27 of the experiment, mice were sacrificed and dissected, and colon tissues were harvested. The **(A)** *Srebf1* and **(B)** *TNF-α* mRNA levels were measured by RT-qPCR. Data are expressed as mean ± standard deviation (SD) (n=6). Compared with the loperamide-induced model mouse group, (***) *p* < 0.001, (****) *p* < 0.0001, compared with the normal control group, (#) *p* < 0.05， (####) *p* < 0.0001, is significant.
